# Comparison of the Whole-Genome Sequence of an Oka Varicella Vaccine from China with Other Oka Vaccine Strains Reveals Sites Putatively Critical for Vaccine Efficacy

**DOI:** 10.1128/JVI.02281-18

**Published:** 2019-04-17

**Authors:** Qiuhua Wu, Pierre Rivailler, Songtao Xu, Wenbo Xu

**Affiliations:** a NHC Key Laboratory of Medical Virology and Viral Diseases (National Institute for Viral Disease Control and Prevention, Chinese Center for Disease Control and Prevention), WHO WPRO Regional Reference Measles/Rubella Laboratory, Beijing, People’s Republic of China; Northwestern University

**Keywords:** VZV, genomics, vaccine

## Abstract

Varicella-zoster virus (VZV) infection results in varicella mostly in children. Reactivation of the virus causes herpes zoster (HZ), mostly in adults. A live attenuated vaccine (vOka-Biken) was originally derived from the parental strain pOka. Several live attenuated vaccines based on the Oka strain are currently available worldwide. In China, varicella vaccines have been licensed by four manufacturers. In this study, we analyze the whole-genome sequence (WGS) of vOka-BK produced by Changchun BCHT Biotechnology also known as Baike. vOka-BK WGS was compared against the genomic sequences of four other Oka strains: pOka, vOka-Biken, vOka-Varilrix from GlaxoSmithKline, and vOka-Varivax from Merck & Co. A previous study identified 137 single nucleotide polymorphisms (SNPs) shared by all vOkas. The current analysis used these data as a reference to compare with vOka-BK WGS and focused on 54 SNPs located in the unique regions of the genome. Twenty-eight nonsynonymous substitutions were identified, ORF62 and ORF55 featuring the most amino acid changes with 9 and 3, respectively. Among the 54 SNPs, 10 had a different mutation profile in vOka-BK compared to the other three vaccines. A comparison with the clade 3 strain Ellen, known to be attenuated, identified three shared amino acid changes: *130R in ORF0 and R958G and S628G in ORF62. This analysis provides the first comparison of a Chinese varicella vaccine to the other vaccines available worldwide and identifies sites potentially critical for VZV vaccine efficacy.

**IMPORTANCE** Varicella, also known as chickenpox, is a highly contagious disease, caused by varicella-zoster virus (VZV). Varicella is a common childhood disease that can be prevented by a live attenuated vaccine. The first available vaccine was derived from the parental Oka strain in Japan in 1974. Several live attenuated vaccines based on the Oka strain are currently available worldwide. Among the four vaccines produced in China, the vaccine manufactured by Changchun BCHT Biotechnology, also known as Baike, has been reported to be very efficacious. Comparative genomic analysis of the Baike vaccine with other Oka vaccine strains identified sites that might be involved in vaccine efficacy, as well as important for the biology of the virus.

## Abstract (precis)

Varicella, also known as chickenpox, is a highly contagious disease, caused by varicella-zoster virus (VZV). Varicella is a common childhood disease that can be prevented by a live attenuated vaccine. The first available vaccine was derived from the parental Oka strain in Japan in 1974. Several live attenuated vaccines based on the Oka strain are currently available worldwide. Among the four vaccines produced in China, the vaccine manufactured by Changchun BCHT Biotechnology, also known as Baike, has been reported to be very efficacious. Comparative genomic analysis of the Baike vaccine with other Oka vaccine strains identified sites that might be involved in vaccine efficacy, as well as important for the biology of the virus.

## INTRODUCTION

**FIG 1:**
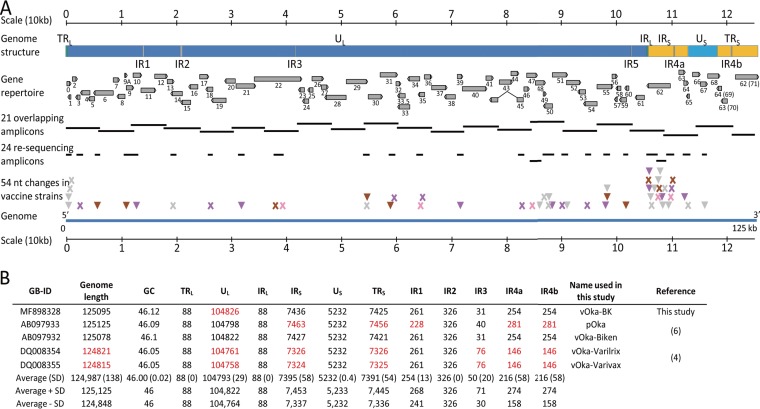
Comparison of VZV genomes. (A) Schematic representation of VZV genome structure and gene repertoire, as well as the sequence strategy used in this report, and the nucleotide variability between parental and vaccine strains. Genomic regions are represented as color-coded boxes: blue for unique regions (dark for U_L_ and light for U_S_), green for repeat long regions (TR_L_ and IR_L_), gray for internal repeats (IR1, IR2, IR3, IR4a, IR4b, and IR5), and orange for repeat short regions (IR_S_ and TR_S_). ORFs are shown as gray arrows, rightward ORFs on the top and leftward ORFs on the bottom. PCR amplicons are shown as thick lines. Fifty-four SNPs in vOka strains compared to pOka are shown as arrowheads for nonsynonymous substitutions and as “X” for synonymous substitutions. The 54 SNPs are color-coded based on the four vOka-BK categories established in this analysis: gray for the vOka-BK positions with the same profile as the three other vaccine strains, purple for the vOka-BK positions with a profile similar to those for vOka-Biken and vOka-Varilrix, pink for vOka-BK positions with profiles similar to that for vOka-Varivax, and brown for vOka-BK positions with a unique profile. (B) Genomic components of the Oka genomes analyzed in this study. Elements outside the range of the average size are indicated in red.

Varicella-zoster virus (VZV; human herpesvirus 3) is a member of the alphaherpesvirus subfamily of the *Herpesviridae* (1). The complete DNA sequence of the VZV genome (Dumas strain) was first determined by Davison and Scott in 1986 (2). VZV has a double-stranded DNA genome of approximately 125 kb with a G+C content of 46%. The genome is composed of a unique long region (U_L_) flanked by inverted repeat regions, namely, the terminal repeat long (TR_L_) and the internal repeat long (IR_L_) sequences, followed by a unique short (U_S_) region flanked by inverted repeat regions, namely, the terminal repeat short (TR_S_) and the internal repeat short (IR_S_) regions (Fig. 1). The genome encodes 70 unique open reading frames (ORFs), three of them encoded within the IR_S_ region (ORFs 62 to 64) being invertedly repeated in the TR_S_ region (ORFs 69 to 71) (1, 2).

VZV is the causative agent of varicella (chickenpox) mostly in children. Subsequently, it establishes latency in the sensory ganglia with the potential to reactivate at a later time to cause herpes zoster (HZ), also known as shingles. Although varicella is usually self-limiting and resolves within several days, severe complications, including pneumonia, encephalitis, congenital infection, or even death, can occur (1).

Varicella can be effectively controlled and prevented by immunization with live attenuated vaccines. Most live attenuated varicella vaccines were derived from the same parental strain (pOka) of wild-type VZV (3, 4). pOka was originally isolated in 1971 from the vesicle ﬂuid of a Japanese child named Oka who had typical chickenpox. The Oka virus was attenuated by sequential passage in cell culture, and the resulting Oka strain vaccine (referred as vOka-Biken in this study) was used to vaccinate Japanese children in 1974 (5). Subsequently, GlaxoSmithKline (GSK) Biologicals and Merck Sharp & Dohme (Merck & Co.) have produced their own formulations of the Oka vaccine, known as Varilrix and Varivax, respectively, which were both derived from the Biken seed stock. Varilrix was first licensed in some European countries in 1984, whereas Varivax was approved in the United States in 1995 (4).

A comparative sequence analysis of pOka and vOka using overlapping PCR and Sanger sequencing revealed 42 single nucleotide polymorphisms (SNPs) between the wild type and the attenuated Oka strain (6). More recently, Depledge et al. completed this analysis by comparing pOka with vOka-Biken, vOka-Varilrix from GSK and vOka-Varivax from Merck & Co. (7). The authors generated whole-genome sequences (WGS) using deep-sequencing technology for one vOka-Biken, two vOka-Varilrix, three vOka-Varivax, and one vOka-Zostavax (related to vOka-Varivax) vaccine preparations. These researchers identified 137 mutated positions present in all vaccine batches. In addition, they showed almost perfect correlation between allele frequencies (AFs) in vOka-Biken and vOka-Varilrix, whereas vOka-Varivax featured significant differences (7).

In China, varicella vaccine became available in 1998. Currently, the vaccine is produced by four domestic companies, namely, Changchun BCHT Biotechnology (also known as Baike), Changchun Keygen Biological Products, Changsheng Biotechnology, and Shanghai Institute of Biologic Products. As with the Merck & Co. and GSK vaccines, all Chinese varicella vaccines were derived from the Biken seed virus. The vaccines are licensed for the susceptible population, those over 12 months old, in China (8). All varicella vaccine productions are based on the seed lot system using classical cell culture methods (9). The immunogenicity and safety of the Chinese varicella vaccine have been previously proved in the marketplace (8–11). Adverse event surveillance has not detected fatalities attributable to vaccine in China, whereas a few varicella fatal cases in immunocompromised patients have been reported in the United States and Germany, and they were shown to be caused by Oka vaccine strain (12, 13).

The present study aimed at analyzing the WGS of the Chinese varicella vaccine produced by Changchun BCHT Biotechnology (also known as Baike). In order to better characterize vOka-BK, vOka-BK WGS was compared to the published sequences of VZV wild-type strains (Dumas and pOka) and vaccine strains (vOka-Biken, vOka-Varilrix, and vOka-Varivax) in the context of Depledge et al. analysis (7).

## RESULTS

### Analysis of vOka-BK WGS.

vOka-BK WGS was generated with 264 sequencing reads of an average of 717 bp and 94% quality score (94% of the base calls have <1% chance of being incorrect). The assembly of an average of 2-fold coverage resulted in a consensus sequence of 125,095 bp (Fig. 1). This genome was characterized by a G+C content of 46.1%, which is a hallmark of VZV genomes (Fig. 1B). As expected, the gene repertoire of vOka-BK was 100% conserved with 70 unique ORFs and 3 ORFs (ORFs 62, 63, and 64) inversely duplicated in the TR_S_ region (Fig. 1A). The overall genome structure of vOka-BK was also conserved, with the sizes of unique and repeated regions similar to what was observed in the genomes of the other vaccine strains (Fig. 1B).

### Comparison of vOka-BK WGS with the genome sequence of the parental strain pOka and 3 vaccine strains, vOka-Biken, vOka-Varilrix, and vOka-Varivax.

vOka-BK WGS (MF898328) was compared to the genome sequences of the parental strain pOka (AB097933) and three vaccine strains vOka-Biken (AB097932), vOka-Varilrix (DQ008354), and vOka-Varivax (DQ008355). A total of 168 nucleotide position differences among the five Oka strains were identified, including the 137 positions previously identified by Depledge et al. (7) (see Table S1). Nearly half of the differences (*n* = 74) were observed in the IR_S_ region, mainly in ORF62. Seventy-two were found in the U_L_ region. The remaining positions were found in the IR_s_ (*n* = 18), IR_L_ (*n* = 3), and U_S_ (*n* = 1) regions. The 18 positions located in the IRs were discarded as similar mutations in the repeat motifs have been previously reported in other VZV genomes with unknown consequences on the biology of the virus. The study by Depledge et al. provided AFs for sequences of vOka-Biken, vOka-Varilrix, and vOka-Varivax genomes (7). Among the 137 SNPs reported by Depledge et al., we chose to exclude 32 sites with an AF of <10% (7). Furthermore, we excluded 54 sites with a low-complexity sequence environment, such as four or more identical nucleotides or dinucleotides repeated multiple times. Finally, we chose to focus on single nucleotide changes. Ten insertions or deletions of one or more nucleotides were detected in noncoding regions, and these changes are not likely to have an effect on the vaccine potential. Among the 54 remaining sites, 47 were within ORFs, and 28 were nonsynonymous substitutions (Fig. 1A, Table 1 , and Table S1). ORF62 and ORF55 featured the most nonsynonymous mutations, with 9 and 3, respectively.

**TABLE 1:**
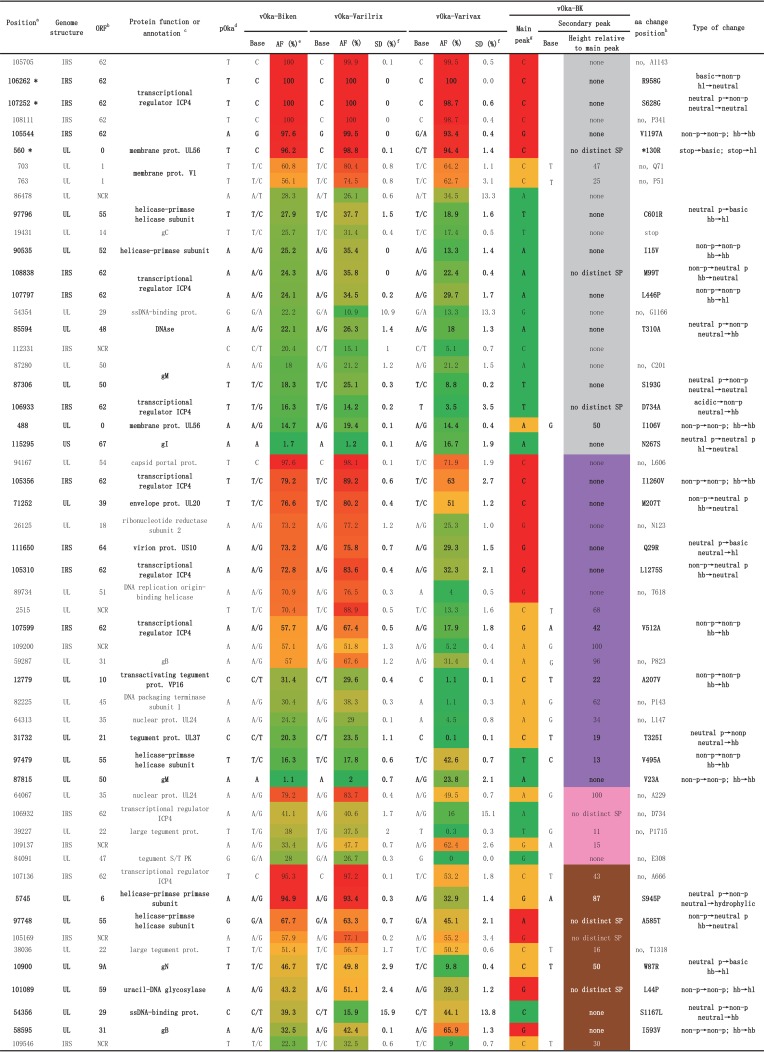
List of 54 SNPs in vOka-Biken, v-Oka-Varilrix, vOka-Varivax, and vOka-BK compared to pOka

*^a^*The 54 SNPs are sorted based on the four vOka-BK categories established in this analysis: gray, vOka-BK positions with the same profile as the three other vaccine strains; purple, vOka-BK positions with profiles similar to those of vOka-Biken and vOka-Varilrix; pink, vOka-BK positions with a profile similar to that of vOka-Varivax; and brown, vOka-BK positions with a unique profile. The positions are also sorted based on the allele frequency of vOka-Biken. Three positions—560, 106262, and 107252—are shared with the clade 3 Ellen strain, known to be attenuated, and are indicated by an asterisk (*) (14).

*^b^*SNPs within ORFs are indicated by the ORF name (number), whereas SNPs outside ORFs are indicated as NCR (noncoding region).

*^c^*Abbreviations: prot., protein; g, glycoprotein; ss, single stranded; S/T PK, serine/threonine protein kinase.

*^d^*The pOka nucleotide is shown, as well as the consensus nucleotide for the three vOkas. Mixed nucleotides are shown, unless the AF is <5% (wild type) or >95% (mutated).

*^e^*Average allele frequencies (AFs) are represented as a heat map: green for wild type (AF = 0%) to red for fixed mutations (AF = 100%) through orange (AF = 50%). Average AF values were computed using raw data from Depledge et al. (7): two vOka-Varilrix vaccine batches on the one hand and three vOka-Varivax batches and one Zostavax batch on the other hand (7). SD, standard deviation.

*^f^*Positions with an SD of >10: AF values for vOka-Varilrix, 54,354 (0%; 21.74%) and 54,356 (0%; 31.82%); AF values for vOka-Varivax, 86,478 (17.24%; 30.40%; 36.15%; 54.17%), 54,354 (0%; 25.64%; 27.55%; 0%), 106,932 (36.16%; 0.96%; 1.84%; 25.11%), and 54,356 (29.41%; 42.24%; 38%; 66.67%). The presented data are based on findings from Depledge et al. (7).

*^g^*The base corresponding to the main peak, as well as to a potential secondary peak, is shown for vOka-BK.

*^h^*SNPs within ORFs are indicated by their amino positions. Nonsynonymous changes are indicated, as well as the type of change in terms of polarity (p, polar) and hydrophobicity (hl, hydrophilic; hb, hydrophobic).

### Comparison of vOka-BK sequence at 54 genomic positions relative to vOka-Biken, vOka-Varilrix, and vOka-Varivax.

vOka-BK assembly was manually checked at each of the 54 positions of interest. In order to increase confidence in the sequence analysis, these 54 positions were checked on four distinct batches, one from 2014 and three from 2018. The three batches from 2018 yielded the same sequence; not only the main peak was identical, but the secondary peak also had a similar height (Table S2). The 2018 sequence of vOka-BK was compared to the sequences of the other three vOka strains. Four profiles were identified relative to the three other vaccine strain sequences: (i) 22 vOka-BK positions featured the same mutation profile as the other vaccine strains (in gray in all figures and tables); (ii) 17 vOka-BK positions featured the same mutation profile as vOka-Biken and vOka-Varilrix (in purple); (iii) 5 vOka-BK positions featured the same mutation profile as vOka-Varivax (in pink); and (iv) 10 vOka-BK positions featured a mutation profile different from the other three vaccine strains (in brown). Eight of these positions were within ORFs, and six were nonsynonymous changes. They were S945P in the primase subunit of helicase primase ORF6, W87R in glycoprotein N (gN) ORF9A, S1167L in the single-stranded DNA-binding protein ORF29, I593V in gB ORF31, A585T in the helicase subunit of the helicase-primase ORF55, and L44P in uracil-DNA glycosylase ORF59.

### Sixteen nucleotide variable positions within the ORF62 region.

**FIG 2:**
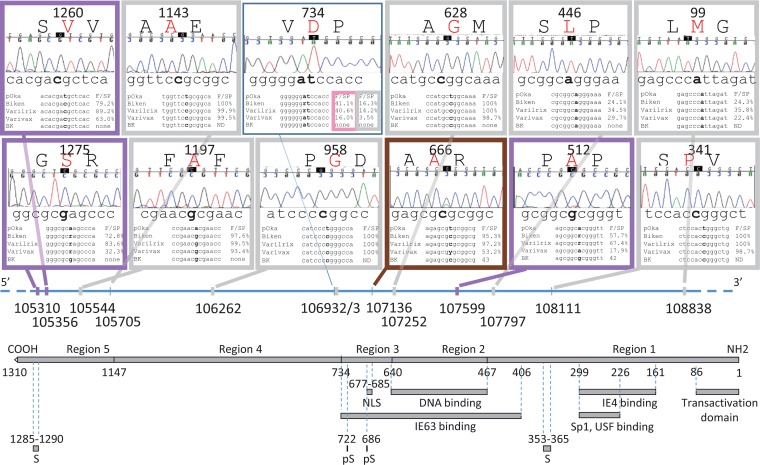
Thirteen SNPs within ORF62. Each SNP is indicated by its genomic position and represented by a box featuring a representative chromatogram for vOka-BK sequencing data, as well as the alignment between pOka, vOka-Biken, vOka-Varilrix, vOka-Varivax, and vOka-BK. The nucleotide of interest is shown in boldface in the alignment. AFs (F) and secondary peaks (SP) are shown for the nucleotide position. A secondary peak below the detection level is indicated as ND. Amino acid residues (in red) and positions within ORF62 are indicated. Except for the box representing positions 106932 and 106933, each box is outlined with a thick line in color based on the four vOka-BK categories established in this analysis: gray for vOka-BK positions with the same profile as the three other vaccine strains, purple for vOka-BK positions with a profile similar to those of vOka-Biken and vOka-Varilrix, pink for vOka-BK positions with a profile similar to that of vOka-Varivax, and brown for vOka-BK positions with a unique profile. For the box corresponding to genomic positions 106932 and 106933, the columns corresponding to allele frequency and secondary peak data are boxed. Positions corresponding to nonsynonymous changes are shown as thick lines on the genome diagram. The bottom of the figure features a functional map drawn on scale based on the review from Ruyechan (35). NLS, nuclear localization signal; IE, immediate early; S, region rich in serine; pS, phosphoserine.

Thirteen SNPs were found within ORF62 resulting in nine nonsynonymous substitutions (Table 1 and Fig. 2). Among them, three positions (107797, 107599, and 107252) were located within functional sites. The corresponding amino acid changes (L446P, V512A, and S628G) were located within the binding site of the transcriptional regulator encoded by ORF63 (Fig. 2). In addition, the mutations V512A and S628G were also located in the DNA binding site encoded within ORF62 protein. Furthermore, two SNPs (109137 and 109200) were found in a putative promoter region of ORF62 gene, located 4 and 137 nucleotides upstream of the ATG (position 109133), respectively. Finally, one SNP (position 105169) was 32 nucleotides downstream of the stop codon (position 105201) and 22 nucleotides upstream of a putative poly(A) motif (105147) (Table 1).

Among the 13 SNPs within the ORF62 region, 8 (105544, 105705, 106262, 106933, 107252, 107797, 108111, and 108838) had a similar profile in all vOkas, including vOka-BK (indicated in gray in Table 1 and Fig. 1 and 2). Five of these positions (105544, 105705, 106262, 107252, and 108111) were fixed or nearly fixed. Among them, the positions 105705 and 108111 did not result in any amino acid change raising the question why such silent nucleotide changes would be selected. The remaining three positions (106933, 107997, and 108838) were mixed with wild-type consensus sequences in all vOkas (AF < 40% or no distinct SP). Two positions (105310 and 105356) were nearly fixed in vOka-Biken and vOka-Varilrix (AF > 70), but vOka-Varivax featured a mixed base at these positions. The vOka-BK profile was similar to that of vOka-Biken and vOka-Varilrix with a mutated base and no significant secondary peak (indicated in purple in Table 1 and Fig. 1 and 2). In the same category, position 107599 was mixed in vOka-Biken and vOka-Varilrix, whereas it was barely mutated in vOka-Varivax. vOka-BK was mixed at this position (shown in purple in Table 1 and Fig. 1 and 2). One position (i.e., 106932) was mixed in vOka-Biken and vOka-Varilrix but was almost wild type (AF = 16%) in vOka-Varivax. Similarly, vOka-Bk also did not show any mutation at this position (indicated in pink in Table 1 and Fig. 1 and 2). Finally, position 107136 had a mutation profile different in vOka-BK compared to the other vOkas (indicated in brown in Table 1 and Fig. 1 and 2). This position was nearly fixed in vOka-Biken and vOka-Varilrix (AF > 95), but it was mixed in vOka-Varivax (AF = 53). vOka-BK position was intermediate and mutated with a distinct secondary peak.

### Nineteen amino substitutions in 15 proteins other than ORF62.

Five substitutions (10900, 58595, 87306, 87815, and 115295) were found in four glycoproteins, including two with a surface exposure, namely, gB (58595, I593V) and gI (115295, N267S), and two glycoproteins involved in the attachment of the virus (Table 2). The other three amino acid substitutions (10900 [W87R] in gN and 87815 [V23A] and 87306 [S193G] in gM) were predicted to be located inside the virion. The rest of the amino substitutions concerned proteins involved in transcription, replication, or egress of the virus and were within undetermined functional domains.

**TABLE 2:**
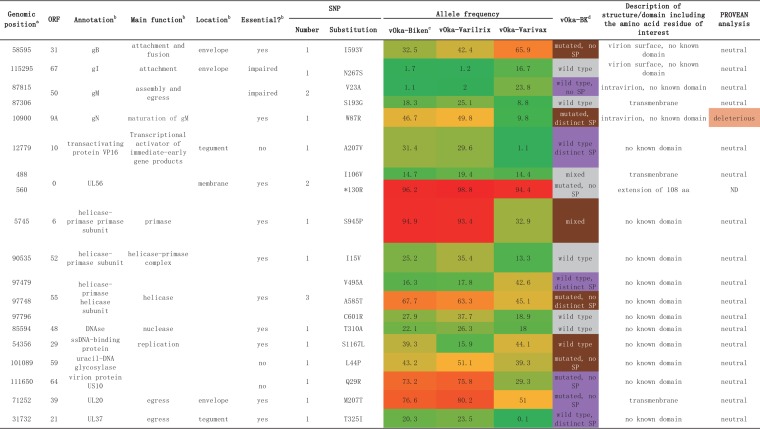
List of 19 nonsynonymous substitutions (excluding ORF62)

*^a^*The proteins are listed based on their role in the biology of the virus, attachment, entry, transcription, replication, and egress.

*^b^*Annotation, main function, location in the virion, and previous results on the role of the protein in the biology of the virus according to Arvin and Gilden (1). g, glycoprotein; ss, single stranded.

*^c^*Average allele frequencies are represented as a heat map, from green for wild type (AF = 0%) to red for fixed mutations (AF = 100%) through orange (AF = 50%). Average AF values were computed using raw data from Depledge et al (7).

*^d^*The status of vOka-BK sequence is indicated according to the four vOka-BK categories established in this analysis: grey for vOka-BK positions with the same profile as the three other vaccine strains, purple for vOka-BK positions with a profile similar to those of vOka-Biken and vOka-Varilrix, and brown for vOka-BK positions with a unique profile.

**FIG 3:**
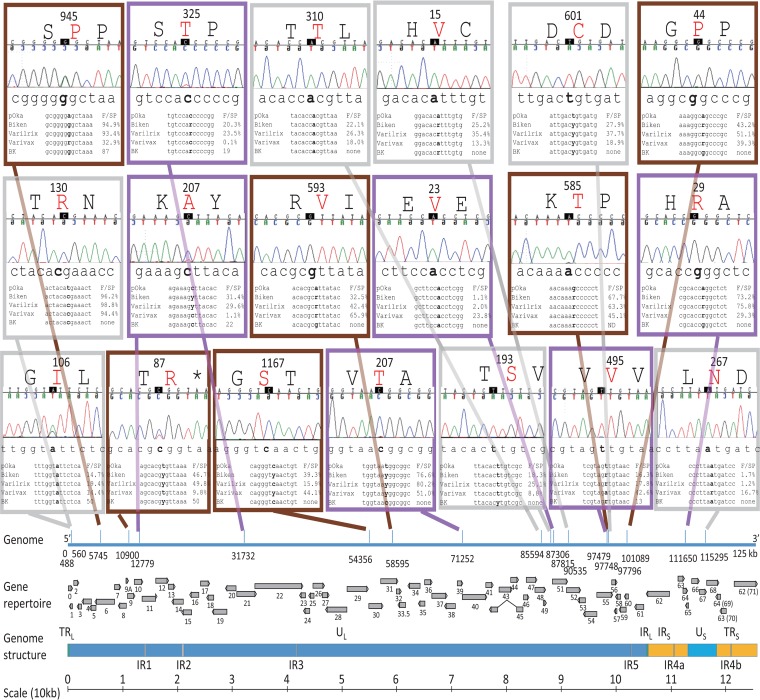
Nineteen nonsynonymous substitutions (excluding ORF62). The layout of the figure is according to Fig. 2. The bottom of the figure features a genomic map in order to better locate the nonsynonymous substitutions within the genome and its ORFs. Genomic regions are represented as color-coded boxes: blue for unique regions (dark for U_L_ and light for U_S_), green for repeat long regions (TR_L_ and IR_L_), gray for internal repeats (IR1, IR2, IR3, IR4a, IR4b, and IR5), and orange for repeat short regions (IR_S_ and TR_S_). ORFs are shown as gray arrows, rightward ORFs on the top and leftward ORFs on the bottom.

Seven positions (488, 560, 85594, 87306, 90535, 97796, and 115295) featured a similar mutation profile in all vOkas, including vOka-BK (indicated in gray in Table 2 and Fig. 1 and 3). One site was particularly intriguing in ORF0. The SNP at position 560 was nearly fixed in all vOkas, including vOka-BK, and resulted in the extension of the protein for an additional 108 amino acids. The remaining six SNPs (488, 85594, 87306, 90535, 97796, and 115295) had similar profiles in all vOkas, being mixed with a wild-type consensus. Six positions (12779, 31732, 71252, 87815, 97479, and 111650) had similar profiles in vOka-Biken, vOka-Varilrix, and vOka-BK (indicated in purple in Table 2 and Fig. 1 and 3). Finally, six positions (5745, 10900, 54356, 58595, 97748, and 101089) had unique mutation profiles in vOka-BK (indicated in brown in Table 2 and Fig. 3). Five of these positions involved proteins that were essential for the biology of the virus. Position 5745, resulting in the amino acid change S945P in the primase ORF6, was nearly fixed in vOka-Biken and vOka-Varilrix, whereas it was mixed with a wild-type consensus in vOka-Varivax. vOka-BK was mixed at this position, with a distinct secondary peak (height at 87). Position 10900, resulting in the amino acid change W87R in gN, was mutated in vOka-BK, but it was mixed in vOka-Biken and vOka-Varilrix and wild type in vOka-Varivax. Position 54356, resulting in the amino acid change S1167L in the DNA binding protein ORF29, was wild type in vOka-BK but mixed in the other vOkas. Position 58595, resulting in the amino acid change I593V in gB, was nearly fixed in vOka-BK, whereas it was mixed in the other three vOkas. Similarly, position 97748, resulting in the amino acid change A585T in the helicase ORF55, was nearly fixed in vOka-BK, whereas it was mixed in the other three vOkas. Finally, position 101089, resulting in the amino acid change L44P in the uracyl DNA-glycosylase ORF59, was mutated in vOka-BK with no distinct secondary peak, whereas it was mixed in the other vOkas. Among the 19 substitutions, only one amino acid change (W87R in ORF9A) was identified as “deleterious” in a PROVEAN analysis (http://provean.jcvi.org/seq_submit.php), meaning that this amino acid substitution was not found in any orthologs (Table 2). Residue 87 is at the very end of the protein, and a change from W to R might not be critical for protein function, even though these residues are chemically very different, i.e., they are neutral hydrophobic and basic hydrophilic, respectively.

## DISCUSSION

VZV vaccine is a live attenuated vaccine. Attenuation was obtained after multiple passages in cultured cells (5). Most of the vaccines were derived from clade 2 strain pOka and passaged at least 30 times in various cell types (4). Comparisons between parental and vaccine genomes identified potential sites that could be associated with attenuation (6, 7). Additional studies on the clade 3 strain Ellen, which became highly attenuated after at least 90 passages in cultured cells, showed that position 560 within ORF0 and positions 106262 and 107252 with ORF62 were likely to be associated with attenuation (14). Attenuation was confirmed using SCID-hu mouse model (15). Infection with vOka and Ellen leads to minimal replication compared to infection with pOka. Mutation at position 560 results in a longer ORF0, 221 amino acids instead of 129. ORF0, also known as ORF S/L, encodes a cytoplasmic protein that might affect adhesion molecules in infected cells (16). More recently, Zhang et al., using the bacterial artificial chromosome technology, showed that the deletion of the entire ORF resulted in a slow-growth phenotype in infected cells (17). Finally, Kaufer et al. demonstrated that ORF0 is essential for VZV replication and could be considered a functional homolog of HSV-1 UL56 (18). Position 107252, corresponding to residue 628 in ORF62 is within the DNA binding site (positions 467 to 640), whereas position 106262 (residue 958) has not been associated with any functional domain of ORF62 (19). Recently, a comparison between WGS from pOka and WGS from seven distinct vOka preparations showed that positions 560, 106262, and 107252 were fixed or nearly fixed (7). The present study confirmed that these positions are mutated in vOka-BK. That these three mutations are shared between two attenuated viruses, vOka and Ellen strain, suggests that these positions might be necessary for the attenuation process. A definite demonstration would be to include these mutations in a wild-type virus and assess whether the recombinant virus is attenuated. Three additional positions within ORF62 are fixed or nearly fixed, namely, 105544, 105705, and 108111. Cohrs et al. showed that transactivation of VZV promoters with ORF62 derived from vOka or pOka were comparable, suggesting that vOka ORF62 was not sufficient to induce attenuation (20). Furthermore, Zerboni et al. showed that attenuation is likely to involve different regions of the genome (21). Comparison of vOka genomes with pOka identified other positions in vOkas that have the same mutation profile, basically mixed with a wild-type consensus. Nine of these nucleotide substitutions resulted in amino acid changes, but none were identified as deleterious in a PROVEAN analysis, suggesting that these amino acid substitutions could be found in other strains. It would, however, be worth assessing whether a fixation of these positions would have an effect on the attenuated phenotype.

Varilrix and Varivax are two of the most used VZV vaccines in the world (4). Even though they are both derived from pOka, a recent study showed that vaccine preparations from Varilrix and Varivax are not genetically identical. Indeed, a correlation of 0.69 was found between vOka-Biken and vOka-Varivax AFs (7). In contrast, Depledge et al. reported an almost perfect correlation between vOka-Biken and vOka-Varilrix AFs (*R*^2^ = 0.98). Varilrix and Varivax have been established after multiple passages in various cell lines, and the discrepancy might be due to passage differences, as reported by Depledge et al. (7). The present study identifies 22 positions that might be related to passage history. Several studies compared Varilrix and Varivax in terms of vaccine efficacy and adverse reaction. Higher seroconversion rates have been reported for Varivax (22, 23). Furthermore, higher risk of varicella breakthrough was reported in patients vaccinated with Varilrix (24). The present study shows that vOka-BK has more sites with a profile like vOka-Biken and vOka-Varilrix. This suggests that vOka-BK might have a vaccine potential similar to those of vOka-Biken and vOka-Varilrix.

Reports concerning Baike vaccine specifically are very scarce. Wang et al. reported a vaccine efficacy of 91% (confidence interval [CI] = 67 to 93%) for Baike vaccine, which is comparable to the average vaccine efficacy (95%; CI = 78 to 84%) computed in a meta-analysis conducted by Marin et al. based on 42 original studies (9, 25). As far as we know, the only publication concerning specifically the Baike vaccine was published in the *Chinese Journal of Immunology* by Dong et al. (26). Briefly, this study concerned 38,883 children from the Binhai New Area in the city of Tianjin, bordering Beijing in the Southeast, aged 12 months to 12 years old, which were monitored from 42 days to 2 years after vaccination with the Baike vaccine. Dong et al. reported 134 breakthrough cases (0.34%). This incidence rate can be compared to the average incidence of 0.85% (CI = 0.53 to 1.37%) computed recently in a meta-analysis of 27 studies by Zhu et al. (27). Thus, reports from Wang et al., as well as by Dong et al., suggest that the Baike vaccine is very efficacious. However, more data are needed, especially for two-dose vaccinations, since the reports mentioned concerned only one-dose vaccinations. In this context, the 10 SNPs that have a different profile in vOka-BK compared to the other Oka vaccine might be involved in a better vaccine efficacy. The present study shows some batch variability at least between years of production. The lots from 2018 seem comparable. In order to have a better sense of vOka-BK vaccine efficacy, it would be necessary to do a side-by-side comparison with the other vOkas.

Currently, many countries have introduced varicella vaccine in a universal routine vaccination (URV) program and administered two doses of vaccine to control and prevent chickenpox (28). The World Health Organization (WHO) recommends varicella vaccine as the URV in the countries and regions where there is a heavy burden of disease. At this time, varicella vaccine is only available in the private sector in China. VZV vaccine has been proved safe and well tolerated (29, 30). Unfortunately, several cases reported that varicella live attenuated vaccine administered to immunocompromised patients could occasionally cause severe adverse events and even death (12, 13). Though very rare, these lethal cases highlight the challenge of a safe administration of live VZV vaccine in immunocompromised patients. It is worth noting that no death has been reported in immunocompromised patients from China. This is likely due to a gap of surveillance, and this highlights the need for better monitoring of varicella outbreaks in China.

In summary, the comparison of the WGS from vOka-BK used in the varicella vaccine preparation manufactured by the Chinese company Changchun BCHT Biotechnology (also known as Baike) to three other vOkas WGS identified three types of SNPs. (i) The first SNP type includes nucleotide positions that feature a similar mutation profile, either mutated or wild type in all vOkas, and thus are likely to be core vaccine positions. A change at these positions might be detrimental to the vaccine’s potential. (ii) The second type of SNP features a different profile in vOka-Varilrix and vOka-Varivax and could be associated with differences in vaccine efficacy. (iii) Finally, the third type of SNP includes nucleotide positions that have a unique profile in vOka-BK that could affect efficacy of the Chinese vaccine.

## MATERIALS AND METHODS

### Vaccine lots and DNA extraction.

vOka-BK was produced by Changchun BCHT Biotechnology (Changchun, China) from vOka-Biken seed stock in MRC5 ATCC cells (5). The number of passages in MRC5 cells is unknown but is likely to be <12 to limit the number of pOka passages to 38, as required by the WHO (31). Four vaccine lots of vOka-BK have been used: one lot produced in 2014 (201402015-1) and three lots produced in 2018 (201802011-2, 201805038-1, and 201805039-1). Whole-genomic DNA was extracted with a QIAamp DNA minikit (Qiagen, Düsseldorf, Germany) according to the manufacturer’s instructions.

### PCR amplification.

Forty-two sequence-specific primers were designed using Sequencher 5.0 software (GeneCodes, Ann Arbor, MI) based on the sequence of the reference strain Dumas (GenBank accession no. X04370) (Fig. 1). Overlapping regions of approximately 200 bases were designed to cover the entire genomic sequence of VZV. Overlapping long PCR fragments (4.0 to 6.5 kb) were amplified with a high-fidelity enzyme mixture of the Platinum *Taq* DNA polymerase high-fidelity kit according to the manufacturer’s instructions (Invitrogen, Carlsbad, CA). The thermal cycler program consisted of an initial hot-start PCR step of 94°C for 2 min, followed by 40 cycles of amplification (94°C for 15 s, 50°C for 30 s, and 68°C for 6.5 min) and a final elongation step at 68°C for 5 min. PCR products were then purified using QIAquick PCR purification and a gel extraction kit (Qiagen) when necessary. In addition, 24 amplicons were designed to specifically cover the 54 genomic positions of interest. PCR fragments (0.8 to 2.0 kb) were amplified with the high-fidelity enzyme mixture of the Platinum *Taq* DNA polymerase high-fidelity kit according to the manufacturer’s instructions. The thermal cycler program was as follows: an initial hot-start PCR step of 94°C for 2 min, followed by 40 cycles of amplification (94°C for 15 s, 50°C for 30 s, and 68°C for 2 min) and a final elongation step at 68°C for 5 min. The sequences of primers used for amplification and sequencing are available upon request.

### DNA sequencing.

Direct sequencing of purified PCR products and plasmid DNA was performed with an ABI Prism dye terminator reaction kit (Perkin-Elmer, Norwalk, CT) and an ABI Prism 3100 genetic analyzer (Applied Biosystems, Hitachi, Japan) according to the manufacturer’s instructions. Sequencing reads were assembled with the Sequencher software version 5.0 (GeneCodes). Positions with ambiguous nucleotides were identified using a default value of 15% for the detection of secondary peaks in Sequencher.

### Cloning of PCR products.

Direct sequencing of the PCR products derived from regions with highly complex secondary structures (flanking regions between IR_L_ and IR_S_ regions), as well as the genomic termini, was complemented by subcloning of amplicons, followed by the sequencing of plasmid clones. Subcloning was also performed when direct sequencing did not generate information of sufficient quality or when a particular SNP could not be reliably confirmed. PCR amplicons were individually cloned by using a TOPO TA cloning kit (Invitrogen, San Diego, CA). Plasmid DNAs were purified from seven bacterial clones with a QIAprep spin kit (Qiagen). DNA sequences of the cloned inserts were determined using vector-specific sequencing primers.

### Sequence analysis.

vOka-BK WGS was compared to the following five genomic sequences from GenBank database (Dumas, X04370; pOka, AB097933; vOka-Biken, AB097932; vOka-Varilrix, DQ008354; and vOka-Varivax, DQ008355). All of the nucleotide and protein positions described in this study are relative to the reference Dumas strain, X04370. Sequences were aligned with MAFFT (32), the SNPs are listed in MEGA6 (33), and sequence alignments were analyzed with BioEdit (v7.0; Tom Hall, North Carolina State University, Raleigh, NC). Genomic maps were generated with Artemis 16.0.0 (34). The average AFs of vOka-Varilrix and vOka-Varivax were computed based on the data reported by Depledge et al. from two GSK (Varilrix) and four Merck & Co. (three Varivax and one Zostavax) vaccine preparations (7).

### Data availability.

Oka-BK WGS is available in GenBank under accession number MF898328.

## Supplementary Material

Supplemental file 1
