# Supplementary material for: Comparison of the Whole-Genome Sequence of an Oka Varicella Vaccine from China with Other Oka Vaccine Strains Reveals Sites Putatively Critical for Vaccine Efficacy
Source: J Virol. 2019 Apr 17;93(9):e02281-18. doi: 10.1128/JVI.02281-18 (PMC6475776; doi:10.1128/JVI.02281-18)
Supplement: Supplemental file 1 [file JVI.02281-18-s0001.pdf]

Comparison of the whole genome sequence of an Oka varicella vaccine from China with other Oka vaccine strains reveals sites putatively critical for vaccine efficacy

Running title (51 characters):

WGS analysis of an Oka varicella vaccine from China

Qiuhua Wu[a], Pierre Rivailler[a], Songtao Xu[a#] and Wenbo Xu[a#]

[a]NHC Key Laboratory of Medical Virology and Viral Diseases (National Institute for Viral Disease Control and Prevention, Chinese Center for Disease Control and Prevention), WHO WPRO Regional Reference Measles/Rubella Laboratory, Beijing 102206, People's Republic of China.

#Corresponding authors: Songtao Xu, xsttz886@sina.com; Wenbo Xu, wenbo_xu1@aliyun.com

Supplementary table 1: List of 168 nucleotide differences among pOka, vOka-Biken, vOka-Varilrix and vOka-Varivax.

Selected for further analysis [1]	Position	in Depledge et al. (137)	in Gomi et al. (41) [2]	Genome structure	pOka	vOka Biken		vOka-Varilrix		vOka-Varivax		vOka-BK			ORF	aa change, position	Type of change	
						Nucleotide	Allele frequency (%) [2]	Nucleotide	Allele frequency (%)	Nucleotide	Allele frequency (%)	Main peak	Secondary peak: Nucleotide	Secondary peak: Height relative to main peak [3]				
low complexity	109	yes		UL	C	C/A	8	C/A	3.1	C/A	15.1	C		none	NCR			
low complexity	178			UL	G	G	NA	C	NA	C	NA	C		none	0	no, A2		
yes	488	yes		UL	A	A/G	14.7	A/G	19.4	A/G	14.4	A	G	50	0	I106V	neutral nonpolar→neutral nonpolar	hydrophobic→hydrophobic
yes	560	yes	yes	UL	T	C	96.2	C	98.8	C	94.4	C		no distinct secondary peak	0	*130R	stop→basic polar	stop→hydrophilic
yes	703	yes	yes	UL	T	T/C	60.8	T/C	80.4	T/C	64.2	C	T	47	1	no, Q71		
yes	763	yes	yes	UL	T	T/C	56.1	T/C	74.5	T/C	62.7	C	T	25	1	no, P51		
low allele frequency	1568	yes		UL	T	T/G	3	T/G	3	T/G	1.7	T		none	2	no, G145		
low complexity	1838			UL	C	G	NA	C	NA	C	NA	C		none	2	no, G235		
yes	2515	yes	yes	UL	T	T/C	70.4	T/C	88.9	T/C	13.3	C	T	68	NCR			
yes	5745	yes	yes	UL	A	A/G	94.9	A/G	93.4	A/G	32.9	G	A	87	6	S945P	neutral polar→neutral nonpolar	hydrophilic→hydrophobic
yes	10900	yes	yes	UL	T	T/C	46.7	T/C	49.8	T/C	9.8	C	T	50	9A	W87R	neutral polar→basic polar	hydrophobic→hydrophilic
low allele frequency	11408	yes		UL	C	C/A	3	C/A	1.9	C	0.4	C		below detection level		P134T	neutral nonpolar→neutral polar	hydrophobic→hydrophilic
yes	12779	yes	yes	UL	C	C/T	31.4	C/T	29.6	C/T	1.1	C	T	22	10	A207V	neutral nonpolar→neutral nonpolar	hydrophobic→hydrophobic
repeat	14242			IR1	as in Dumas	Insertion CGCGATCGA CGACGAGG GAGAGGCG GAGGAGGA		as in Dumas		as in Dumas		as in Dumas		NA	11			
low allele frequency	17247	yes		UL	T	T/G	0.8	T	0.4	T	0.2	T		none		V345G	neutral nonpolar→neutral nonpolar	hydrophobic→NA
yes	19431	yes	yes	UL	T	T/C	25.7	T/C	31.4	T/C	17.4	T		none	14	stop		
repeat	20703			IR2	C	C	NA	C/T	NA	C	NA	C	T	18	14	no, K137		
repeat	20711			IR2	T	T	NA	A	NA	A	NA	T		ND	14	S135T	neutral polar →neutral polar	hydrophilic→hydrophilic
repeat	20745			IR2	A	A	NA	C	NA	C	NA	A		none	14	K123N	basic polar→neutral polar	hydrophilic→hydrophilic
repeat	20787			IR2	A	A	NA	C/A	NA	C	NA	A		none	14	K109N	basic polar→neutral polar	hydrophilic→hydrophilic
repeat	20829			IR2	A	A	NA	C/A	NA	C/A	NA	A		none	14	K95N	basic polar→neutral polar	hydrophilic→hydrophilic
repeat	20837			IR2	T	T	NA	A/T	NA	A/T	NA	T		none	14	T93S	neutral polar →neutral polar	hydrophilic→hydrophilic
repeat	20871			IR2	C	C	NA	C/A	NA	C/A	NA	C		none	14	K81N	basic polar→neutral polar	hydrophilic→hydrophilic
repeat	20879			IR2	A	A	NA	A/T	NA	A	NA	A		none	14	S79T	neutral polar →neutral polar	hydrophilic→hydrophilic
repeat	20913			IR2	C	C	NA	A/C	NA	A/C	NA	C		none	14	K67N	basic polar→neutral polar	hydrophilic→hydrophilic
yes	26125	yes	yes	UL	A	A/G	73.2	A/G	77.2	A/G	25.3	G		none	18	no, N123		
yes	31732	yes	yes	UL	C	C/T	20.3	C/T	23.5	C	0.1	C	T	19	21	T325I	neutral polar→neutral nonpolar	hydrophilic→hydrophobic
low allele frequency	37126	yes		UL	A	A/C	14.1	A/C	11.1	A/C	3.1	A		no distinct secondary peak	22	E1015A	acidic polar→neutral nonpolar	NA→hydrophobic
yes	38036	yes	yes	UL	T	T/C	51.4	T/C	56.7	T/C	50.2	C	T	16	22	no, T1318		
yes	39227	yes	yes	UL	T	T/G	38	T/G	37.5	T	0.3	T	G	11	22	no, P1715		
low allele frequency	39527	yes		UL	A	A/G	8.9	A/G	7.7	A/G	1.8	A		none	22	no, G1815		
low complexity	40413	yes		UL	G	G/T	4.7	G/T	1.3	G/T	1	G		none	22	A2111S	neutral nonpolar→neutral polar	hydrophobic→hydrophilic
low allele frequency	41298	yes		UL	T	T/C	10.8	T/C	8.5	T/C	4.5	T		no distinct secondary peak	22	C2406R	neutral polar→basic polar	NA→hydrophilic
repeat	41476			IR3	T	C		T		T		gap		NA	22	A→V	neutral nonpolar→neutral nonpolar	hydrophobic→hydrophobic
repeat	41476			IR3	C	G		C		C		gap		NA	22	A→V	neutral nonpolar→neutral nonpolar	hydrophobic→hydrophobic
repeat	41485			IR3	C	C		T		C		gap		NA	22	A→V	neutral nonpolar→neutral nonpolar	hydrophobic→hydrophobic
repeat	41494			IR3	C	C/T		C/T		C		gap		NA	22	A→V	neutral nonpolar→neutral nonpolar	hydrophobic→hydrophobic
repeat	41499			IR3	A	A		C		C		gap		NA	22	T→P	neutral polar→neutral nonpolar	hydrophilic→hydrophobic
low complexity	42387	yes		UL	T	T/A	2.3	T/A	3.1	T/A	1	T		none	NCR			
low complexity	42390	yes		UL	A	A/G	1.3	A	0.9	A	0.4	A		none	NCR			
NCR	42403			UL	Deletion	Deletion 3As		Insertion A		Deletion A		Deletion 2As		NA	NCR			
low complexity	42404	yes		UL	C	C/A	5	C/A	4	C/A	2.7	C		none	NCR			
low complexity	42407	yes		UL	C	C/A	1.1	C	0.7	C	0.2	C		none	NCR			
low allele frequency	42714	yes		UL	G	G/A	2.9	G/A	3.7	G	0.3	G		none	23	A142V	neutral nonpolar→neutral nonpolar	hydrophobic→hydrophobic
low allele frequency	43629	yes		UL	A	A/G	8.9	A/G	9.8	A/G	3.1	A		none	24	no, S131		
low complexity	53787	yes		UL	T	T/C	10.1	T/C	11.2	T/C	1.6	T		none	29	no, R977		
yes	54354	yes		UL	G	G/A	22.2	G/A	10.9	G/A	13.3	G		none	29	no, G1166		
yes	54356	yes		UL	C	C/T	39.3	C/T	15.9	C/T	44.1	C		none	29	S1167L	neutral polar→neutral nonpolar	hydrophilic→hydrophobic
low allele frequency	57051	yes		UL	A	A/T	1.2	A/T	1.8	A	0.3	A		none	31	Y78F	neutral polar→neutral nonpolar	hydrophobic→hydrophobic
low allele frequency	57080	yes		UL	T	T/G	5.5	T/G	5.3	T	0.6	T		below detection level	31	S88A	neutral polar→neutral nonpolar	hydrophilic→hydrophobic
yes	58595	yes	yes	UL	A	A/G	32.5	A/G	42.4	A/G	65.9	G		none	31	I593V	neutral nonpolar→neutral nonpolar	hydrophobic→hydrophobic
yes	59287	yes	yes	UL	A	A/G	57	A/G	67.6	A/G	31.4	G	A	96	31	no, P823		
low complexity	60267	yes		UL	A	A/C	6.6	A/C	8.6	A/C	1.7	A		no distinct secondary peak	NCR			
low complexity	60268	yes		UL	A	A	0.8	A	0.4	A/C	1.6	A		no distinct secondary peak	NCR			

Supplementary table 1: List of 168 nucleotide differences among pOka, vOka-Biken, vOka-Varilrix and vOka-Varivax.

Selected for further analysis [1]	Position	in Depledge et al. (137)	in Gomi et al. (41) [2]	Genome structure	pOka	vOka Biken		vOka-Varilrix		vOka-Varivax		vOka-BK			ORF	aa change, position	Type of change	
						Nucleotide	Allele frequency (%) [2]	Nucleotide	Allele frequency (%)	Nucleotide	Allele frequency (%)	Main peak	Secondary peak Nucleotide	Secondary peak Height relative to main peak [3]				
NCR	60278	yes		UL	Deletion	Deletion 3As		Deletion A		Deletion 2As		Deletion A		NA	NCR			
low complexity	60281	yes		UL	C	C/A	3.2	C/A	4.4	C/A	2.8	C		none	NCR			
yes	64067	yes	yes	UL	A	A/G	79.2	A/G	83.7	A/G	49.5	G	A	100	35	no, A229		
yes	64313	yes		UL	A	A/G	24.2	A/G	29	A/G	4.5	A	G	34	35	no, L147		
low complexity	67417	yes		UL	T	T/G	7.4	T/G	6.9	T/G	4.3	T	G	66	37	N448K	neutral polar→basic polar	hydrophilic→hydrophilic
low complexity	69850	yes		UL	T	T/G	5.3	T/G	3.3	T/G	1.6	T		ND	38	no, T148		
yes	71252	yes	yes	UL	T	T/C	76.6	T/C	80.2	T/C	51	C		none	39	M207T	neutral polar→neutral polar	hydrophobic→hydrophilic
NCR	78144			UL	Deletion T	Deletion T		Deletion T		Deletion T		as in Dumas		NA	NCR			
yes	82225	yes	yes	UL	A	A/G	30.4	A/G	38.3	A	1.1	A	G	62	45	no, P143		
low allele frequency	83774	yes		UL	C	C/A	1	C/A	1.2	C	0.1	C		below detection level		L203I	neutral nonpolar→neutral nonpolar	hydrophobic→hydrophobic
yes	84091		yes	UL	G	G/A	28	G/A	26.7	G	0	G		no distinct secondary peak	47	no, E308		
yes	85594	yes		UL	A	A/G	22.1	A/G	26.3	A/G	18	A		none	48	T310A	neutral polar→neutral nonpolar	hydrophilic→hydrophobic
yes	86478	yes		UL	A	A/T	28.3	A/T	26.1	A/T	34.5	A		none	NCR			
yes	87280	yes	yes	UL	A	A/G	18	A/G	21.2	A/G	21.2	G		none	50	no, C201		
yes	87306	yes	yes	UL	T	T/C	18.3	T/C	25.1	T/C	8.8	T		none	50	S193G	neutral polar→neutral nonpolar	hydrophilic→NA
yes	87815	yes		UL	A	A	1.1	A	2	A/G	23.8	A		none	50	V23A	neutral nonpolar→neutral nonpolar	hydrophobic→hydrophobic
yes	89734	yes	yes	UL	A	A/G	70.9	A/G	76.5	A/G	4	G		none	51	no, T618		
yes	90535	yes	yes	UL	A	A/G	25.2	A/G	35.4	A/G	13.3	G		none	52	I15V	neutral nonpolar→neutral nonpolar	hydrophobic→hydrophobic
low allele frequency	92209	yes		UL	A	A/G	1	A/G	1.3	A/G	4	A		below detection level		T573P	neutral polar→neutral nonpolar	hydrophilic→hydrophobic
low allele frequency	93569	yes		UL	G	G/A	2	G/A	1.3	G	0.3	G		below detection level	53	no, H94		
yes	94167	yes	yes	UL	T	T/C	97.6	T/C	98.1	T/C	71.9	C		none	54	no, L606		
yes	97479	yes		UL	T	T/C	16.3	T/C	17.8	T/C	42.6	T	C	13	55	V495A	neutral nonpolar→neutral nonpolar	hydrophobic→hydrophobic
yes	97748	yes	yes	UL	G	G/A	67.7	G/A	63.3	G/A	45.1	A		no distinct secondary peak	55	A585T	neutral nonpolar→neutral polar	hydrophobic→hydrophilic
yes	97796	yes	yes	UL	T	T/C	27.9	T/C	37.7	T/C	18.9	T		none	55	C601R	neutral polar→basic polar	NA→hydrophilic
low complexity	98694	yes		UL	G	G/T	0.9	G	0.6	G	0.3	G		none		V43F	neutral nonpolar→neutral nonpolar	hydrophobic→hydrophobic
low complexity	99906	yes		UL	T	T/C	4.8	T/C	6.1	T/C	1.8	C	T	30		K123E	basic polar→acidic polar	hydrophilic→NA
yes	101089	yes	yes	UL	A	A/G	43.2	A/G	51.1	A/G	39.3	G		no distinct secondary peak	59	L44P	neutral nonpolar→neutral nonpolar	hydrophobic→hydrophobic
low complexity	101616	yes		UL	A	A/C	0.9	A	0.7	A	0.4	A		below detection level	60	F12V	neutral nonpolar→neutral nonpolar	hydrophobic→hydrophobic
low complexity	101617	yes		UL	A	A/T	0.9	A	0.7	A	0.4	A		below detection level	60	no, V11		
low allele frequency	101624	yes		UL	T	T/A	1.4	T/A	1.5	T/A	1.1	T		no distinct secondary peak	60	Q9L	neutral polar→neutral nonpolar	hydrophilic→hydrophobic
low allele frequency	101625	yes		UL	G	G	0.7	G/T	1	G	0.9	G		below detection level	60	Q9K	neutral polar→basic polar	hydrophilic→hydrophilic
low allele frequency	102019	yes		UL	G	G/A	0.9	G	0.8	G	0.6	G		no distinct secondary peak	NCR			
repeat	102219			IR5	as in Dumas	as in Dumas		Insertion,TC		same			as in Dumas	NA	NCR			
low allele frequency	102670	yes		UL	A	A/G	10.8	A/G	12	A/G	2.9	A		ND	NCR			
NCR	104980			IRL	as in Dumas	as in Dumas		as in Dumas		as in Dumas		Insertion T		NA	NCR			
low complexity	104981	yes		IRL	C	C/T	2.4	C/T	1.7	C	0.7	C		no distinct secondary peak	NCR			
low complexity	105010	yes		IRL	C	C/G	82.9	C/G	84.6	C/G	82.1	G		no distinct secondary peak	NCR			
NCR	105020			IRS	Insertion, C	as in Dumas		Insertion, C		Insertion, C		as in Dumas		NA	NCR			
low complexity	105021	yes		IRS	G	G/C	7.1	G/C	12	G/C	19.1	G		no distinct secondary peak	NCR			
low complexity	105022	yes		IRS	G	G/C	4.2	G/C	4.3	G/C	6.9	G		below detection level	NCR			
low complexity	105046	yes		IRS	G	G/C	9.1	G/C	11.1	G/C	8	G		below detection level	NCR			
low complexity	105056	yes		IRS	T	T/A	4.6	T/A	10	T/A	3.6	T		none	NCR			
low complexity	105057	yes		IRS	A	A/T	5.1	A/T	7.7	A/T	3.4	A		no distinct secondary peak	NCR			
low complexity	105063	yes		IRS	G	G/C	28.2	G/C	38.5	G/C	23.5	G		below detection level	NCR			
low complexity	105152	yes		IRS	C	C	0.9	C	0.4	C/A	3.1	C		below detection level	NCR			
low complexity	105153	yes		IRS	A	A	0.7	A	0.4	A/C	2.9	A		none	NCR			
yes	105169	yes	yes	IRS	A	A/G	57.9	A/G	77.1	A/G	55.2	G		below detection level	NCR			

Supplementary table 1: List of 168 nucleotide differences among pOka, vOka-Biken, vOka-Varilrix and vOka-Varivax.

Selected for further analysis [1]	Position	in Depledge et al. (137)	in Gomi et al. (41) [2]	Genome structure	pOka	vOka Biken		vOka-Varilrix		vOka-Varivax		vOka-BK			ORF	aa change, position	Type of change	
						Nucleotide	Allele frequency (%) [2]	Nucleotide	Allele frequency (%)	Nucleotide	Allele frequency (%)	Main peak	Secondary peak Nucleotide	Height relative to main peak [3]				
low allele frequency	105179	yes		IRS	A	A/G	2.7	A/G	2.8	A/G	10	A		none	NCR			
yes	105310	yes	yes	IRS	A	A/G	72.8	A/G	83.6	A/G	32.3	G	A	none	62	L1275S	neutral nonpolar→neutral polar	hydrophobic→hydrophilic
yes	105356	yes	yes	IRS	T	T/C	79.2	T/C	89.2	T/C	63	C		none	62	I1260V	neutral nonpolar→neutral nonpolar	hydrophobic→hydrophobic
low complexity	105391	yes		IRS	T	T/C	0.9	T	0.9	T	0.2	T		below detection level	62	D1248G	acidic polar→neutral nonpolar	NA→NA
yes	105544	yes	yes	IRS	A	G	97.6	G	99.5	G	93.4	G		none	62	V1197A	neutral nonpolar→neutral nonpolar	hydrophobic→hydrophobic
low complexity	105567	yes		IRS	C	C/G	2	C/G	1.8	C/G	1	C		below detection level	62	E1189D	acidic polar→acidic polar	NA→NA
low allele frequency	105679	yes		IRS	G	G/T	1.5	G/T	1.2	G/T	5.2	G	T	12	62	A1152D	neutral nonpolar→acidic polar	hydrophobic→NA
yes	105705	yes	yes	IRS	T	C	100	C	99.9	C	99.5	C		none	62	no, A1143		
yes	106262	yes	yes	IRS	T	C	100	C	100	C	100	C		none	62	R958G	basic polar→neutral nonpolar	hydrophilic→NA
low allele frequency	106650	yes		IRS	T	T/C	1.7	T/C	2.2	T/C	2.7	T		below detection level	62	no, R828		
low allele frequency	106831	yes		IRS	T	T/C	0.3	T	0.2	T	0.9	T		below detection level	62	Q768R	neutral polar→basic polar	hydrophilic→hydrophilic
yes	106932	yes		IRS	A	A/G	41.1	A/G	40.6	A/G	16	A		no distinct secondary peak	62	no, D734		
yes	106933	yes		IRS	T	T/G	16.3	T/G	14.2	T/G	3.5	T		no distinct secondary peak	62	D734A	acidic polar→neutral nonpolar	NA→hydrophobic
low complexity	106934	yes		IRS	C	C/G	1.3	C/G	1.9	C	0.3	C		below detection level	62	D734H	acidic polar→basic polar	NA→hydrophilic
low complexity	106936	yes		IRS	A	A/C	4.3	A/C	3.7	A/C	1.4	A		below detection level	62	V733G	neutral nonpolar→neutral nonpolar	hydrophobic→NA
low complexity	106939	yes		IRS	A	A/C	3.7	A/C	2.8	A	0.4	A		below detection level	62	V732G	neutral nonpolar→neutral nonpolar	hydrophobic→NA
low allele frequency	106949	yes		IRS	A	A/G	1.1	A/G	1.9	A	0.3	A		none	62	S729P	neutral polar→neutral nonpolar	hydrophilic→hydrophobic
low allele frequency	106972	yes		IRS	A	A/C	2.3	A/C	2	A/C	1	A		none	62	V721G	neutral nonpolar→neutral nonpolar	hydrophobic→NA
yes	107136	yes	yes	IRS	T	T/C	95.3	T/C	97.2	T/C	53.2	C	T	43	62	no, A666		
yes	107252	yes	yes	IRS	T	C	100	C	100	C	98.7	C		none	62	S628G	neutral polar →neutral nonpolar	hydrophilic→NA
low complexity	107428	yes		IRS	G	G/C	2.4	G/C	1.4	G	0.2	G		none	62	A569G	neutral nonpolar→neutral nonpolar	hydrophobic→NA
low complexity	107432	yes		IRS	G	G/C	0.5	G	0.7	G	0.1	G		none	62	R568G	basic polar→neutral nonpolar	hydrophilic→NA
yes	107599	yes	yes	IRS	A	A/G	57.7	A/G	67.4	A/G	17.9	G	A	42	62	V512A	neutral nonpolar→neutral nonpolar	hydrophobic→hydrophobic
yes	107797	yes	yes	IRS	A	A/G	24.1	A/G	34.5	A/G	29.7	A		none	62	L446P	neutral nonpolar→neutral nonpolar	hydrophobic→hydrophobic
low allele frequency	108030	yes		IRS	T	T/C	1.7	T/C	2	T/C	4.1	T		none	62	E368A	acidic polar→neutral nonpolar	NA→hydrophobic
yes	108111	yes	yes	IRS	T	C	100	C	100	C	98.7	C		none	62	no, P341		
yes	108838	yes	yes	IRS	A	A/G	24.3	A/G	35.8	A/G	22.4	A	G	no distinct secondary peak	62	M99T	neutral polar →neutral polar	hydrophobic→hydrophilic
low allele frequency	109007	yes		IRS	T	T/C	0.4	T	0.9	T	0.8	T		none	62	S43G	neutral polar→neutral nonpolar	hydrophilic→NA
low allele frequency	109035	yes		IRS	G	G/T	1.1	G/T	1.2	G/T	6.6	G		none	62	no, A33		
yes	109137	yes	yes	IRS	A	A/G	33.4	A/G	47.7	A/G	62.4	G	A	15	NCR			
yes	109200	yes	yes	IRS	A	A/G	57.1	A/G	51.8	A/G	5.2	G	A	100	NCR			
yes	109546	yes		IRS	T	T/C	22.3	T/C	32.5	T/C	9	C	T	30	NCR			
low allele frequency	109697	yes		IRS	G	G/C	2	G/C	1.9	G/C	4.9	G		none	NCR			
low allele frequency	109698	yes		IRS	T	T/A	1.3	T/A	1.4	T/A	3.6	T		none	NCR			
low allele frequency	109699	yes		IRS	C	C/T	1.3	C/T	1	C/T	2.7	C		none	NCR			
low allele frequency	109700	yes		IRS	A	A/G	1	A	0.8	A/G	2.6	A		none	NCR			
low allele frequency	109701	yes		IRS	G	G	0.8	G	0.6	G/T	1.9	G		none	NCR			
low allele frequency	109702	yes		IRS	G	G	0.9	G	0.4	G/C	1.5	G		none	NCR			
low complexity	109759	yes		IRS	A	A/C	3.1	A/C	2.8	A/C	1.2	A		none	NCR			
NCR	109907			IRS	as in Dumas	as in Dumas		Insertion,GG		same		as in Dumas		NA	NCR			
NCR	109907			IRS	Insertion,G	Insertion,GGG		as in Dumas		as in Dumas		same as		NA	NCR			
low complexity	110020	yes		IRS	T	T/G	1.8	T	0.8	T	0.5	T		no distinct secondary peak	NCR			
low complexity	110030	yes		IRS	T	T	9.4	T/G	10.3	T	4.7	T		no distinct secondary peak	NCR			
low complexity	110035	yes		IRS	T	T/G	13.1	T/G	11.8	T/G	10.4	T		no distinct secondary peak	NCR			
low complexity	110043	yes		IRS	T	T/G	5.8	T/G	5.5	T/G	6.7	T		no distinct secondary peak	NCR			
NCR	110212			IRS	Deletion AT	as in Dumas		as in Dumas		Deletion AT		Deletion AT		NA	NCR			
low complexity	110214			IRS	T	T	NA	T	NA	G	NA	G		none	NCR			
NCR	110214			IRS	Insertion, ATATAG	as in Dumas		as in Dumas		as in Dumas		as in Dumas		NA	NCR			
low complexity	110370	yes		IRS	C	C/A	8.5	C/A	12.3	C/A	22.2	C		no distinct	NCR			
low complexity	110371	yes		IRS	A	A/C	2.2	A/C	3.3	A	0.5	A		no distinct secondary peak	NCR			

Supplementary table 1: List of 168 nucleotide differences among pOka, vOka-Biken, vOka-Varilrix and vOka-Varivax.

Selected for further analysis [1]	Position	in Depledge et al. (137)	in Gomi et al. (41) [2]	Genome structure	pOka	vOka Biken		vOka-Varilrix		vOka-Varivax		vOka-BK			ORF	aa change, position	Type of change	
						Nucleotide	Allele frequency (%) [2]	Nucleotide	Allele frequency (%)	Nucleotide	Allele frequency (%)	Main peak	Secondary peak Nucleotide	Secondary peak Height relative to main peak [3]				
low complexity	110372	yes		IRS	C	C/A	1.6	C/A	3.1	C/A	2.4	C		no distinct secondary peak	NCR			
low complexity	110373	yes		IRS	A	A/C	2	A/C	3.5	A	0.7	A		no distinct secondary peak	NCR			
low complexity	110375	yes		IRS	G	G		G		G/A	11.3	G		no distinct secondary peak	NCR			
low complexity	110376	yes		IRS	G	G		G		G/C	15.3	G		no distinct secondary peak	NCR			
low complexity	110379	yes		IRS	C	C/G	20.5	C/G	22.3	C/G	55.8	C		no distinct secondary peak	NCR			
low complexity	110385	yes		IRS	C	C	0.8	C/T	1	C/T	1.8	C		no distinct secondary peak	NCR			
yes	111650	yes	yes	IRS	A	A/G	73.2	A/G	75.8	A/G	29.3	G		none	64	Q29R	neutral polar →basic polar	hydrophilic→hydrophilic
low complexity	112125	yes		IRS	T	T/A	1.5	T/A	9.6	T/A	8.3	T		none	NCR			
NCR	112128			IRS	Deletion A	Deletion A		Insertion		Insertion A		Insertion 5As		NA	NCR			
low complexity	112138	yes		IRS	T	T/A	5	T/A	9.3	T/A	5	T		none	NCR			
low complexity	112142	yes		IRS	T	T/A	1.8	T/A	5.6	T/A	1.9	T		none	NCR			
low complexity	112143	yes		IRS	T	T/A	1.2	T/A	4.1	T/A	1.9	T		none	NCR			
low complexity	112149	yes		IRS	C	C/T	1.4	C/T	2.4	C	0.7	C		none	NCR			
yes	112331	yes		IRS	C	C/T	20.4	C/T	15.1	C/T	5.1	C		none	NCR			
yes	115295	yes		US	A	A/G	1.7	A/G	1.2	A/G	16.7	A		none	67	N267S	neutral polar→neutral polar	hydrophilic→hydrophilic
repeat	41475–83			IR3	Deletion, GCGCAGC CC	as in Dumas		same deletion		same deletion			as in Dumas	NA	22			
repeat	41484–519			IR3	as in Dumas	as in Dumas		Deletion,GC GCAGCCC GCGCAGA CCGTCCA GCCCGCG CAGCCC		same deletion			as in Dumas	NA	22	del AQPAQTVQPAQP		

1- 54 positions have been selected. The reason for no selection is indicated (low allele frequency, low complexity, repeat).

2- Gomi et al. reported 42 sites but position 106710 does not show any change in Depledge et al studies. Therefore, this site has not been considered.

3- Average allele frequency values were computed using raw data from Depledge et al, 2 vOka-Varilrix vaccine batches on one hand, 3 vOka-Varivax batches and 1 Zostavax bacth on the other hand (Depledge et al., J.Virol.,2016).

4- ND: Not determined

Supplementary Table 2: 54 SNPs in 4 lots of vOka-BK

Year of production	2014				2018											
Lot number	201402015-1				201802011-2				201805038-1				201805039-1			
			Secondary peak				Secondary peak				Secondary peak				Secondary peak	
Position	Number of reads	Main peak [1]	Base	Height relative to main peak [1]	Number of reads	Main peak	Base	Height relative to main peak	Number of reads	Main peak	Base	Height relative to main peak	Number of reads	Main peak	Base	Height relative to main peak
488	2	A	G	12	2	A	G	50	2	A	G	78	2	A	G	58
560	1	C		no distinct SP	2	C		none	2	C		no distinct SP	2	C		no distinct SP
703	2	C	T	12	2	C	T	47	2	C	T	38	2	C	T	34
763	1	C		no distinct SP	2	C	T	25	2	C	T	23	2	C	T	15
2515	2	C		no distinct SP	2	C	T	68	2	C	T	62	2	C	T	65
5745	2	G		none	2	G	A	87	2	G	A	96	2	G	A	90
10900	1	C	T	39	1	C	T	50	1	C	T	50	1	C	T	52
12779	1	C		none	1	C	T	22	1	C	T	31	1	C	T	25
19431	1	T	C	26	2	T		none	2	T		none	2	T		none
26125	1	G	A	12	3	G		none	2	G		none	2	G		none
31732	2	C	T	20	2	C	T	19	2	C	T	15	2	C	T	14
38036	1	C	T	50	2	C	T	16	1	C	T	18	1	C	T	10
39227	1	T	G	100	2	T	G	11	1	T	G	19	1	T	G	10
54354	2	G		no distinct SP	2	G		none	2	G		none	2	G		none
54356	2	C		no distinct SP	2	C		none	2	C		none	2	C		none
58595	1	G		no distinct SP	2	G		none	2	G		none	2	G		none
59287	1	G		no distinct SP	2	A	G	96	2	A	G	94	2	A	G	93
64067	1	G		none	2	G	A	100	2	G	A	100	2	G	A	100
64313	2	A	G	15	2	A	G	34	2	A	G	33	2	A	G	34
71252	2	C		none	2	C		none	2	C		none	2	C		none
82225	1	A	G	35	2	A	G	62	2	A	G	48	2	A	G	60
84091	1	G		no distinct SP	1	G		none	1	G		none	1	G		none
85594	2	A	G	13	2	A		none	1	A		none	1	A		none
86478	2	A		none	1	A		none	1	A		none	4	A		none
87280	3	G	A	30	2	A		none	3	A		none	7	A		none
87306	2	T	C	74	3	T		none	3	T		none	12	T		none
87815	2	A		none	1	A		none	1	A		none	4	A		none
89734	1	G		none	1	G		none	1	G		none	1	G		none
90535	2	G	A	56	2	A		none	2	A		none	2	A		none
94167	1	C		none	2	C		none	2	C		none	2	C		none
97479	1	T	C	42	2	T	C	13	2	T	C	13	2	T	C	13
97748	2	A	G	21	2	A	G	11	2	A	G	9	2	A	G	9
97796	1	T	C	17	2	T		none	2	T		none	2	T		none
101089	2	G		no distinct SP	2	G		none	2	G		none	2	G	A	13
105169	1	G		no distinct SP	1	G		none	2	G		none	1	G		no distinct SP
105310	1	G	A	8	1	G		none	2	G		none	1	G		none
105356	1	C		none	1	C		none	2	C		none	1	C		none
105544	2	G		none	2	G		none	2	G		none	2	G		none
105705	1	C		no distinct SP	2	C		none	1	C		none	1	C		none
106262	1	C		no distinct SP	2	C		none	1	C		none	2	C		none
106932	1	A		none	2	A		none	5	A		none	1	A		none
106933	1	T		none	2	T		none	5	T		none	1	T		none
107136	1	C		none	2	C	T	43	5	C	C	39	1	C	T	31
107252	1	C		none	3	C		none	5	C		none	1	C		none
107599	2	G		none	3	G	A	42	4	G	A	35	2	G	A	29
107797	2	A		none	2	A		none	3	A		none	2	A		none
108111	1	C		no distinct SP	1	C		none	1	C		none	1	C		none
108838	2	A	G	22	2	A		none	2	A		none	2	A		none
109137	2	G	A	19	2	G	A	15	2	G	A	16	2	G	A	14
109200	2	G	A	16	2	A	G	100	2	A	G	90	2	A	G	100
109546	2	C	T	37	2	C	T	30	2	C	T	37	2	C	T	27
111650	2	G		none	2	G		none	1	G		none	1	G		none
112331	1	C	T	76	1	C		none	1	C		none	1	C		none
115295	1	A	G	11	2	A		none	2	A		none	2	A		none

1- Discrepancies between 2014 and 2018 lots are shown in red or pink. Red when the 2014 mutation profile is different from 2018 data (main peak different or mutated vs wild type), pink when the 2014 mutation profile is comparable but not similar to 2018 data (distinct SP vs none).
